# Preoperative predictors of adverse pathological outcomes following radical cystectomy for muscle-invasive bladder cancer in a tertiary referral center

**DOI:** 10.25122/jml-2026-0088

**Published:** 2026-07

**Authors:** Matei Maximilian Buzoianu, Iulia Andras, Lorin Giurgiu, Paul Medan, Stefana Medan, Andrei Popa, Emanuel Cata, Emanuela Ionutas, Marius Apetrei, Ioan Coman, Nicolae Crisan

**Affiliations:** 1 Urology Department, Iuliu Hatieganu University of Medicine and Pharmacy, Cluj-Napoca, Romania

**Keywords:** radical cystectomy, non-organ confined disease, lymphovascular invasion

## Abstract

Accurate preoperative identification of non-organ-confined disease in patients with muscle-invasive bladder cancer could guide patient selection for bladder-sparing strategies. We evaluated the association between available preoperative clinical variables linked to worse oncologic outcomes and pathologic upstaging (≥T3) at radical cystectomy in patients with muscle-invasive bladder cancer. We reviewed our cohort of patients with clinical T2 bladder cancer who underwent radical cystectomy at the Municipal Hospital of Cluj-Napoca, Romania, between 2017 and 2026. Univariable and multivariable logistic regression analyses were used to evaluate the association between preoperative clinical variables: tumor size > 3 cm at transurethral resection (TUR), chronic kidney disease (CKD), hydronephrosis, lymphovascular invasion (LVI), and non-organ-confined disease (≥pT3) at radical cystectomy. The final cohort consisted of 129 patients who underwent radical cystectomy and were included in the analysis. Lymphovascular invasion was independently associated with non-organ-confined disease on multivariable analysis across the entire cohort (OR = 6.99; 95% CI, 2.83–18.5; *P* < 0.001), among patients who received neoadjuvant chemotherapy (OR = 10.5; 95% CI, 2.62–53.1; *P* = 0.002), and among those who did not (OR = 5.13; 95% CI, 1.41–21.2; *P* = 0.017). Hydronephrosis was associated with non-organ-confined disease on univariable analysis but did not remain significant on multivariable analysis in the overall cohort (OR = 1.77; 95% CI, 0.71–4.36; *P* = 0.2). Chronic kidney disease and tumor size greater than 3 cm were associated with non-organ-confined disease on univariable but not multivariable analysis. Lymphovascular invasion was the most consistent independent predictor of pathologic upstaging at radical cystectomy. Further research in larger cohorts is needed to validate these findings.

## Introduction

Muscle-invasive bladder cancer (MIBC) remains one of the most challenging malignancies in uro-oncology, accounting for approximately 25% of newly diagnosed bladder cancer cases. Radical cystectomy with pelvic lymph node dissection remains the established gold-standard treatment for localized disease [1]. Nevertheless, growing evidence supports bladder-preserving multimodal strategies incorporating maximal transurethral resection of the bladder tumor (TURBT) with concurrent chemotherapy and radiotherapy as safe and effective alternatives in carefully selected patients with favorable oncologic characteristics [2].

Identifying optimal candidates for bladder preservation, however, remains a major challenge. Clinical staging of MIBC is inherently imperfect and relies primarily on pathological assessment of TURBT specimens and cross-sectional imaging, including computed tomography and multiparametric magnetic resonance imaging. Despite these approaches, accurately identifying patients harboring non-organ-confined disease, such as ≥pT3 tumors or lymph node metastases, remains difficult. This limitation is clinically relevant, as patients with advanced pathological stage experience inferior oncologic outcomes and should benefit from a more radical treatment [3].

Accordingly, a major limitation of bladder-preserving approaches is the limited ability to accurately identify patients most likely to benefit from this treatment strategy. Evidence regarding the prognostic value of readily available clinical variables for predicting adverse pathological outcomes after radical cystectomy remains limited and often conflicting. Nevertheless, several preoperative parameters have been proposed as surrogates of aggressive disease biology. Preoperative hydronephrosis has consistently been associated with locally advanced tumors and inferior survival outcomes. Similarly, chronic kidney disease (CKD) may reflect advanced disease burden, urinary tract obstruction, or impaired physiological reserve. Tumor size greater than 3 cm at transurethral resection (TUR) has likewise been linked to high-risk disease and worse oncologic outcomes. Lymphovascular invasion (LVI) identified on TURBT specimens has also been associated with adverse pathological features and worse oncologic outcomes following radical cystectomy [4].

We hypothesized that preoperative hydronephrosis, chronic kidney disease, tumor size greater than 3 cm at TUR, and lymphovascular invasion could each serve as predictors of adverse pathology at radical cystectomy, defined as pathological stage ≥pT3 disease. To our knowledge, data regarding the independent predictive value of these factors remain limited and heterogeneous. Identifying such variables associated with adverse pathological outcomes could inform patient counseling and help identify individuals who may benefit from intensified treatment approaches. Therefore, this study aimed to evaluate the independent association between routinely available preoperative clinical variables and adverse pathological outcomes following radical cystectomy.

## Material and Methods

We reviewed our cohort of patients with clinically staged T2 bladder cancer who underwent radical cystectomy at the Municipal Clinical Hospital of Cluj-Napoca, Romania, between 2017 and 2026. Patients with metastatic disease as well as concomitant upper tract urothelial carcinoma were excluded from the analysis. The study included patients with clinical stage T2 disease who underwent TURBT at our institution, as well as patients referred from external institutions for definitive surgical treatment. Clinical staging was determined using TURBT findings together with preoperative imaging, including either a CT urogram or multiparametric bladder MRI for local staging and a chest CT scan to assess metastatic disease. Patients who underwent radical cystectomy for high-risk non-muscle-invasive bladder cancer were excluded from the analysis. All patients included had pathological assessment of lymphovascular invasion status performed on either the initial TURBT specimen or a subsequent transurethral resection specimen. Patients with missing data were excluded. All patients undergoing radical cystectomy underwent standard pelvic lymph node dissection, which included the removal of obturator nodes and internal and external iliac nodes. Patients who received neoadjuvant chemotherapy were treated with either gemcitabine plus cisplatin or the MVAC regimen (methotrexate, vinblastine, doxorubicin, and cisplatin).

The primary outcome of this study was to evaluate the association between preoperative clinical variables and non-organ-confined disease on radical cystectomy specimens, defined as pathological stage T3 or higher. Because patients receiving neoadjuvant chemotherapy generally have a more favorable oncologic prognosis and may experience pathologic downstaging at radical cystectomy, we also analyzed this subgroup, as well as those who did not receive neoadjuvant chemotherapy. We assigned tumor stage according to the 2017 American Joint Committee on Cancer TNM classification. We performed univariable and multivariable logistic regression analyses to evaluate the association between clinical variables and oncologic outcomes.

We performed an exploratory analysis of the association between these variables and pathologically positive lymph nodes on radical cystectomy specimens (pN+). Given the small number of positive lymph nodes in our cohort, we performed only univariable analysis.

Several clinical variables have been reported in the literature to be associated with worse oncologic outcomes after radical cystectomy, including progression under intravesical bacillus Calmette–Guérin (BCG) therapy, variant histology, concomitant carcinoma in situ, clinical stage, tumor size greater than 3 cm at TUR, chronic kidney disease, preoperative hydronephrosis, and lymphovascular invasion identified on transurethral resection specimens. Given the relatively small number of events in our cohort, we performed an exploratory analysis to identify variables with the strongest univariable association with non-organ-confined disease and restricted the multivariable model to these variables to preserve statistical power. The preoperative variables retained for the final analysis were tumor size greater than 3 cm at TUR, chronic kidney disease, preoperative hydronephrosis, and lymphovascular invasion identified on transurethral resection specimens. All analyses were conducted using R version 4.5.0 with the tidyverse (v2.0.0) and gtsummary (v2.2.0) packages [5].

## Results

A total of 147 patients were identified, of whom four patients underwent radical cystectomy for high-risk non-muscle invasive bladder cancer, five declined surgery, and nine opted for radiotherapy. The final cohort consisted of 129 patients who underwent robotic, laparoscopic, or open radical cystectomy, with the type of urinary diversion selected based on disease characteristics and patient comorbidities. The median age was 66 years, and 78% of patients were male. Forty-three patients had stage III or higher chronic kidney disease (eGFR <60 mL/min/1.73 m^2^), and 80 patients (62%) received neoadjuvant chemotherapy. Baseline patient characteristics are summarized in Table 1.

**Table 1:** Baseline characteristics of patients with muscle-invasive bladder cancer who underwent radical cystectomy between 2017 and 2026

Characteristic	No neoadjuvant chemotherapy *n* = 49	Neoadjuvant chemotherapy *n* = 80
Age	67 (63–73)	65 (59–68)
Male	35 (71%)	65 (81%)
BMI	26.4 (22.7–29.2)	28.1 (24.9–30.9)
Progression under BCG	7 (15%)	10 (13%)
Tumor >3 cm at TUR	35 (76%)	55 (72%)
Secondary histology at TUR	6 (12%)	4 (5%)
Preoperative eGFR	69 (46, 85)	68 (51, 85)
Preoperative hydronephrosis	24 (49%)	33 (41%)
**Urinary diversion**		
Cutaneous ureterostomy	26 (53%)	36 (46%)
Ileal conduit	19 (39%)	35 (45%)
Neobladder	4 (8.2%)	7 (9%)
**T stage**		
T0	0	13 (17%)
T1	11 (22%)	17 (22%)
T2	15 (31%)	16 (21%)
T3/T4	23 (47%)	31 (40%)
No. of lymph nodes	10 (7, 14)	11 (8, 14)
N+	10 (22%)	14 (21%)

Data are median (IQR) or *n* (%). Percentages are column percentages. BCG, bacillus Calmette–Guérin; BMI, body mass index; eGFR, estimated glomerular filtration rate; IQR, interquartile range; TUR, transurethral resection.

Among the entire cohort, CKD (OR = 3.55; 95% CI, 1.69–7.68; *P* = 0.001), tumor size greater than 3 cm (OR = 2.56; 95% CI, 1.09–6.39; *P* = 0.035), hydronephrosis (OR = 3.34; 95% CI, 1.62–7.04; *P* = 0.001), and LVI (OR = 7.86; 95% CI, 3.52–18.6; *P* < 0.001) were associated with pathologic upstaging at radical cystectomy on univariable analysis. On multivariable analysis, only LVI remained an independent predictor of pathologic upstaging (OR = 6.99; 95% CI, 2.83–18.5; *P* < 0.001), while CKD (OR = 1.43; 95% CI, 0.54–3.76; *P* = 0.5), tumor size greater than 3 cm (OR = 2.08; 95% CI, 0.75–6.14; *P* = 0.2), and hydronephrosis (OR = 1.77; 95% CI, 0.71–4.36; *P* = 0.2) were no longer significant (Table 2).

**Table 2:** Univariable and multivariable analyses of predictors of non–organ-confined disease at radical cystectomy

	Univariable analysis			Multivariable analysis		
Characteristic	OR	95% CI	*P* value	OR	95% CI	*P* value
CKD	3.55	1.69–7.68	0.001	1.43	0.54–3.76	0.5
Tumor >3 cm	2.56	1.09–6.39	0.035	2.08	0.75–6.14	0.2
Hydronephrosis	3.34	1.62–7.04	0.001	1.77	0.71–4.36	0.2
Lymphovascular invasion	7.86	3.52–18.6	<0.001	6.99	2.83–18.5	<0.001

Data are odds ratios from logistic regression with 95% CIs. CI, confidence interval; CKD, chronic kidney disease; OR, odds ratio.

When the cohort was stratified by receipt of neoadjuvant chemotherapy (NAC), similar trends were observed but with some differences in significance, likely reflecting the smaller subgroup sizes. Among patients who received neoadjuvant chemotherapy (NAC) (*n* = 80), hydronephrosis (OR = 6.48; 95% CI, 2.46–18.2; *P* < 0.001) and LVI (OR = 14.5; 95% CI, 4.53–57.7; *P* < 0.001) were significantly associated with non-organ-confined disease on univariable analysis, as was CKD (OR = 3.24; 95% CI, 1.24–8.78; *P* = 0.018). Tumor size greater than 3 cm was not significantly associated with pathologic upstaging on univariable analysis (OR = 2.08; 95% CI, 0.73–6.58; *P* = 0.2). On multivariable analysis, LVI remained the only independent predictor of pathologic upstaging (OR = 10.5; 95% CI, 2.62–53.1; *P* = 0.002; Table 3).

**Table 3:** Univariable and multivariable analyses of predictors of non–organ-confined disease at radical cystectomy in patients who received neoadjuvant chemotherapy

	Univariable analysis			Multivariable analysis		
Characteristic	OR	95% CI	*P* value	OR	95% CI	*P* value
CKD	3.24	1.24–8.78	0.018	0.73	0.16–2.92	0.7
Tumor >3 cm	2.08	0.73–6.58	0.2	1.88	0.50–7.86	0.4
Hydronephrosis	6.48	2.46–18.2	<0.001	3.11	0.91–10.9	0.070
Lymphovascular invasion	14.5	4.53–57.7	<0.001	10.5	2.62–53.1	0.002

Data are odds ratios from logistic regression with 95% CIs. CI, confidence interval; CKD, chronic kidney disease; OR, odds ratio.

Among patients who did not receive NAC (*n* = 49), LVI was the only variable significantly associated with pathologic upstaging on both univariable (OR = 4.22; 95% CI, 1.32–14.7; *P* = 0.018) and multivariable analysis (OR = 5.13; 95% CI, 1.41–21.2; *P* = 0.017). Although CKD (univariable OR = 3.06; 95% CI, 0.92–11.0; *P* = 0.074; multivariable OR = 2.48; 95% CI, 0.58–11.7; *P* = 0.2) and tumor size greater than 3 cm (univariable OR = 3.56; 95% CI, 0.87–18.4; *P* = 0.094; multivariable OR = 2.91; 95% CI, 0.55–19.7; *P* = 0.2) were associated with an increased risk of pathologic upstaging, but without reaching statistical significance. Hydronephrosis was not associated with pathologic upstaging in this subgroup on either univariable (OR = 1.27; 95% CI, 0.41–3.97; *P* = 0.7) or multivariable analysis (OR = 0.79; 95% CI, 0.18–3.23; *P* = 0.7; Table 4).

**Table 4:** Univariable and multivariable analyses of predictors of non–organ-confined disease at radical cystectomy in patients who did not receive neoadjuvant chemotherapy

	Univariable analysis			Multivariable analysis		
Characteristic	OR	95% CI	*P* value	OR	95% CI	*P* value
CKD	3.06	0.92–11.0	0.074	2.48	0.58–11.7	0.2
Hydronephrosis	1.27	0.41–3.97	0.7	0.79	0.18–3.23	0.7
Lymphovascular invasion	4.22	1.32–14.7	0.018	5.13	1.41–21.2	0.017
Tumor >3 cm	3.56	0.87–18.4	0.094	2.91	0.55–19.7	0.2

Data are odds ratios from logistic regression with 95% CIs. CI, confidence interval; CKD, chronic kidney disease; OR, odds ratio.

Progression for non-muscle-invasive bladder cancer treated with BCG was associated with worse oncologic outcomes. Given the low number of such cases in our cohort, we performed an additional univariable analysis on the entire cohort, but no association with pathologic upstaging was observed (OR = 1.26; 95% CI, 0.44–3.52; *P* = 0.7).

On exploratory univariable analysis of pathologically positive lymph nodes (pN+), CKD (OR = 4.02; 95% CI, 1.59–10.6; *P* = 0.004) and lymphovascular invasion (OR = 13.1; 95% CI, 4.64–43.7; *P* < 0.001) were significantly associated with pN+ disease, while tumor size greater than 3 cm (OR = 3.11; 95% CI, 0.96–14.0, *P* = 0.087) and hydronephrosis (OR = 1.82; 95% CI, 0.74–4.60; *P* = 0.2) were not (Table 5).

**Table 5:** Univariable predictors of pathological lymph node involvement

Characteristic	OR	95% CI	*P* value
CKD	4.02	1.59–10.6	0.004
Tumor >3 cm	3.11	0.96–14.0	0.087
Hydronephrosis	1.82	0.74–4.60	0.2
Lymphovascular invasion	13.1	4.64–43.7	<0.001

Data are odds ratios from univariable logistic regression with 95% CIs. CI, confidence interval; CKD, chronic kidney disease; OR, odds ratio.

## Discussion

We found that lymphovascular invasion was independently associated with pathologic upstaging at radical cystectomy on multivariable analysis, both across the entire cohort and within each treatment subgroup. Preoperative hydronephrosis was associated with non-organ-confined disease on univariable analysis, but this association did not persist on multivariable analysis in the overall cohort. Chronic kidney disease and tumor size greater than 3 cm were similarly associated with pathologic upstaging on univariable but not multivariable analysis. On exploratory univariable analysis, lymphovascular invasion and chronic kidney disease were also associated with positive lymph nodes. These findings are consistent with previously reported data in the literature.

The association between lymphovascular invasion on the transurethral resection specimen and adverse oncologic outcomes is consistent with the findings of Werntz *et al.*, who demonstrated that LVI identified on TURBT independently predicted adverse pathology at radical cystectomy, including pathologic upstaging (OR = 3.7; 95% CI, 1.7–8.0) and lymph node-positive disease (OR = 4.9; 95% CI, 2.1–11.1) [6]. The wide confidence intervals observed in our analysis are attributable to the relatively small cohort size. Dulf *et al*. similarly reported that LVI was associated with more advanced disease and significantly worse progression-free survival (hazard ratio [HR] = 4.85; 95% CI, 2.19–10.77; *P* < 0.001) [7]. In a recent systematic review including 78,107 patients, Mari *et al*. found that LVI was associated with disease recurrence (pooled HR 1.57; 95% CI, 1.45–1.70) and cancer-specific mortality (CSM) (pooled HR = 1.59; 95% CI, 1.48–1.73) in all studies regardless of tumor stage and node status (pT1-4 pN0-2) [8].

Chronic kidney disease has traditionally been considered a marker of worse oncologic outcomes in patients with muscle-invasive bladder cancer. The underlying rationale is that impaired renal function frequently precludes the use of cisplatin-based neoadjuvant chemotherapy, which is known to improve overall survival in this setting. In our cohort, CKD was consistently associated with pathologic upstaging on univariable analysis but was not an independent predictor on multivariable analysis in either the overall cohort or any treatment subgroup. In our cohort, 43 patients had CKD (eGFR <60 mL/min/1.73 m^2^), of whom 33% did not receive neoadjuvant chemotherapy. Supporting our findings, a study of 1,214 patients with CKD and muscle-invasive bladder cancer who underwent radical cystectomy demonstrated that worse renal function was independently associated with adverse pathology (≥pT3 or node-positive disease) on multivariable analysis (OR = 6.96; 95% CI, 3.20–15.12) [9].

Preoperative hydronephrosis has traditionally been considered a strong clinical predictor of non-organ-confined disease, as it can reflect intramural or extravesical tumor infiltration, or compression of the ureter by locally advanced tumor. In our cohort, hydronephrosis was associated with an increased risk of pathologic upstaging on univariable analysis, but this did not remain significant on multivariable analysis in the overall cohort, and it was not associated with an increased risk of nodal disease. In a study by Bartsch *et al*., patients with hydronephrosis had a 70% risk of non-organ-confined disease and a 40% risk of lymph node metastases; on multivariable analysis, hydronephrosis was independently associated with worse recurrence-free survival at a median follow-up of 35 months [10]. In contrast, in El-Adawy’s cohort of 196 patients, preoperative hydronephrosis was not associated with pathologic upstaging (*P* = 0.24) [11]. Larger cohorts are needed to confirm the role of hydronephrosis in predicting adverse pathology.

Tumor size greater than 3 cm at TUR is recognized by the European Association of Urology guidelines as a marker of high-risk disease, given its association with worse oncologic outcomes. In a multicenter study by Soria *et al*., among patients with T1G3 bladder cancer initially managed with BCG who subsequently progressed and underwent radical cystectomy, those with tumors ≥3 cm at diagnosis had a shorter time to cystectomy for locally advanced (≥pT3) or node-positive disease compared with those with smaller tumors (HR = 1.71; 95% CI, 1.22–2.41; *P* = 0.002) [12]. In our cohort, tumor size greater than 3 cm at the time of TURBT was associated with an increased risk of non-organ-confined disease on univariable analysis in the overall cohort (OR = 2.56; 95% CI 1.09–6.39; *P* = 0.035). However, this association was not significant in the multivariable model and was not observed in analyses stratified by neoadjuvant chemotherapy status.

From a clinical standpoint, the consistent independent association between lymphovascular invasion and non-organ-confined disease across the overall cohort and both treatment subgroups suggests that LVI status on TURBT specimens may represent a valuable factor in treatment decision-making and patient counseling regarding the suitability of bladder-preserving strategies versus radical cystectomy.

This study has several limitations that warrant consideration. First, the relatively small sample size limits the generalizability of our findings, and larger, prospective cohorts will be required to validate these results. Second, the CKD threshold used (eGFR <60 mL/min/1.73 m^2^) may underestimate the clinical impact of renal impairment, as many such patients remain eligible for cisplatin-based neoadjuvant chemotherapy and may therefore carry a more favorable prognosis than those with more severely impaired renal function. Third, given the limited number of events in our cohort, the multivariable model was restricted to the four variables with the strongest univariable association with non-organ-confined disease; other clinically relevant factors, such as variant histology, concomitant carcinoma in situ, clinical stage, and progression under intravesical BCG, could not be incorporated into the multivariable analysis without risking model overfitting, and their independent contribution to adverse pathology could therefore not be assessed. Despite these limitations, our findings underscore that some preoperative clinical variables may predict adverse pathology at radical cystectomy. Further research is warranted to validate these results.

## Conclusion

Lymphovascular invasion was the most consistent independent predictor of non-organ-confined disease at radical cystectomy and may aid treatment selection between bladder-preserving approaches and radical cystectomy. Validation in larger cohorts is warranted to confirm these findings.

## Data Availability

The datasets generated and/or analyzed during the current study are available from the corresponding author on request.
